# A focus on phosphinophosphination of apolar bonds by a structurally constrained P–P bonded system

**DOI:** 10.1039/d4sc90251c

**Published:** 2025-01-06

**Authors:** Tyler J. Hannah, Saurabh S. Chitnis

**Affiliations:** a Department of Chemistry, Dalhousie University 6243 Alumni Crescent Halifax Nova Scotia B3H 4R2 Canada tyler.hannah@dal.ca saurabh.chitnis@dal.ca

## Abstract

In this article, we highlight the recent report of Greb *et al.* on the use of a structurally constrained P–P bonded system for phosphinophosphination of alkenes, alkynes, and carbonyls with high regio- and stereoselectivity (https://doi.org/10.1039/D4SC06581F).

## Abstract 2

This article highlights recent work by Greb *et al.* on the phosphinophosphination of alkenes, alkynes, and carbonyls, with a focus on how the polarity of the P–P bond controls the scope and resulting reactivity.

## Graphical abstract

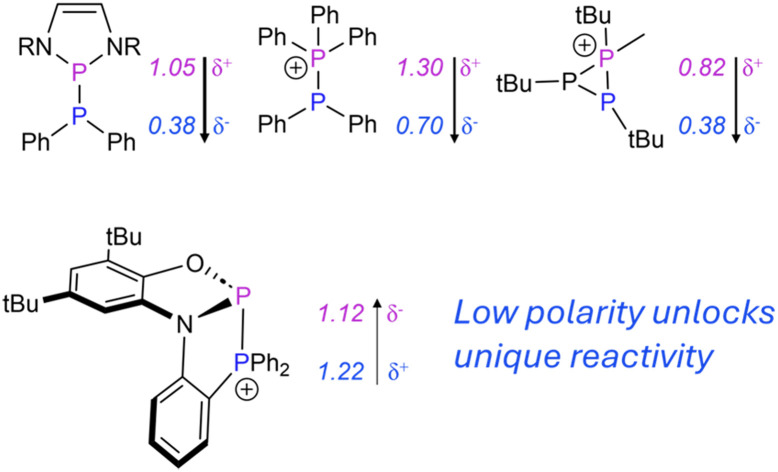

Functionalization of organic substrates by main-group systems is a rapidly advancing field with the potential to complement or exceed established transition metal chemistry for valuable transformations.^1^ An emergent strategy in this pursuit is the use of multi-dentate ligands with p-block elements. Detailed studies involving pnictogen complexes have shown that such ligands can distort molecules to expose vacant orbitals for coordination and template the ensuing activation steps by enforcing the proximity of reactive centres.^2–7^

Cation 1, reported by Greb *et al.*,^8^ is in some ways similar to known intramolecularly-stablized phospheniums, whose high electrophilicity and geometrically-constrained nature allow activation of various E–H (E = N, C, H, O) bonds *via* a stepwise mechanism.^9–14^ But due to their very polar P–X (X = O, N, C) bonds, these stabilized phospheniums are not well-suited to activate weakly-polar or non-polar π-systems. In contrast, 1 features a homoatomic P–P bond that readily adds to non-activated alkynes, alkenes, and carbonyls. This diphosphination reaction was shown to proceed through a concerted mechanism, which enabled high regio- and diastereoselectivity (Scheme 1). Furthermore, this system demonstrates the first diphosphination of an unactivated C=C double bond. Interestingly, 1 exhibits low reactivity towards very polar alkenes, setting the stage for selective transformations when multiple reactive groups are present. The products of phosphinophosphination, 2 and 3, are 7-membered phosphaheterocycles that are in most cases obtained with a high preference for one set of racemic diastereomers. These compounds are of interest for their photophysical properties, which were demonstrated by absorption in the visible region for a derivative of 2. Additionally, a derivative of 3 was able to coordinate to a rhodium complex, demonstrating an approach to access unusual chiral phosphine ligands.

**Scheme 1:**
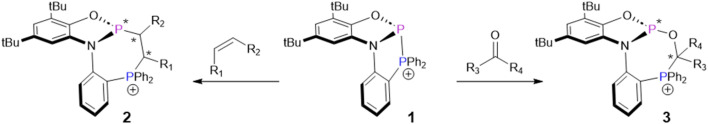
Diphosphination of alkenes and carbonyls by compound 1. Asterisks denote newly formed chiral centres. Counterions have been omitted for simplicity.

The unprecedented reactivity and selectivity of 1 can be understood *via* a closer look at the P–P bond in this cation relative to other phosphino–phosphoniums. Scheme 2 shows the calculated partial charges on phosphorus in 1 and other P–P bonded systems that have been utilized for diphosphination. Compound A features a very polarized P–P bond despite being neutral. This is due to strong resonance stabilization of the phosphenium ion resulting from P–P heterolysis and the attachment to two electronegative nitrogen atoms at phosphorus. The very polar bond in this species is able to react with polar substrates including nitriles and alkenes containing one electron withdrawing group.^15^ Compound B, with a slightly less polar P–P bond, is able to react with activated terminal alkynes through a frustrated Lewis pair mechanism, but only under forcing conditions.^16^ The intramolecular phosphino–phosphonium C features a less polar P–P bond than B, but due to the strained three-membered ring, it can nevertheless diphosphinate some polar substrates such as nitriles, while no addition across less polar unsaturated groups was reported.^17,18^ Although phosphino–phosphoniums, as in B and C, have been shown to have similar barriers for both homolytic and heterolytic bond cleavage, their reactivity is dominated by polar, stepwise addition due to the considerable bond polarization.^19,20^

**Scheme 2:**
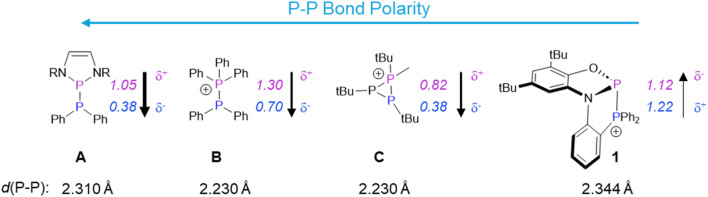
P–P bonded systems utilized for diphosphination and a comparison of their P–P bond polarities and lengths with those of 1.^21,22^ Calculated partial charges are denoted in italics. These values have been taken from the relevant literature.^18,8,23^ Counterions have been omitted for simplicity. For C, only the bond length involving the phosphino–phosphonium unit is given.

Compared to A–C, compound 1 features a minimally polarized P–P bond: the high charge at its donor phosphorus is comparable to the values for B and C, and the high charge at its acceptor phosphorus arises from attachment to σ-withdrawing, π-donating groups (as in the case of A). The π-donor groups on the acceptor phosphorus also result in a very long P–P bond (even longer than that of A). This unique combination of a long and low-polarity P–P bond is well-suited for weak or non-polar π-bond activation *via* a concerted pathway that is not available to polar (A) or strongly-bonded (B, C) analogues. As a bonus, the lack of epimerizable intermediates in a concerted mechanism results in the stereoselectivity observed in transformations starting from 1. The thermodynamic driving force is provided by replacement of a weak P–P bond and a strong C=C or C=O bond with two strong P–C (or P–O) bonds.

The multidenticity of the ligand is also a key enabling feature of this system. Indeed, the P–P bond in 1 is very unusual, since phosphenium cations with strongly π-donating N-substituents do not generally coordinate to triarylphosphines. They are instead readily isolated as stable two-coordinate species.^24,25^ In 1, the pincer ligand reinforces the bonding interaction between the two phosphorus centres to generate a functional group that may not persist if the interaction were intermolecular. In this manner, the chemistry of 1 is also reminiscent of intramolecular frustrated Lewis pairs.^26^ Besides templating the P–P interaction, the ligand constraint is also expected to lower the energy of the disphenoidal transition state (TS_conc_) of the concerted addition pathway, as planarization of three out of four substituents is facilitated by their tethering (Fig. 1).^27^ Finally, the use of a weakly-coordinating [Al(OC(CF_3_)_3_)_4_] counterion ensures that poor donors such as non-polar π-systems can out-compete the anion for coordination to the cation, enabling the subsequent diphosphination.

**Fig. 1:**
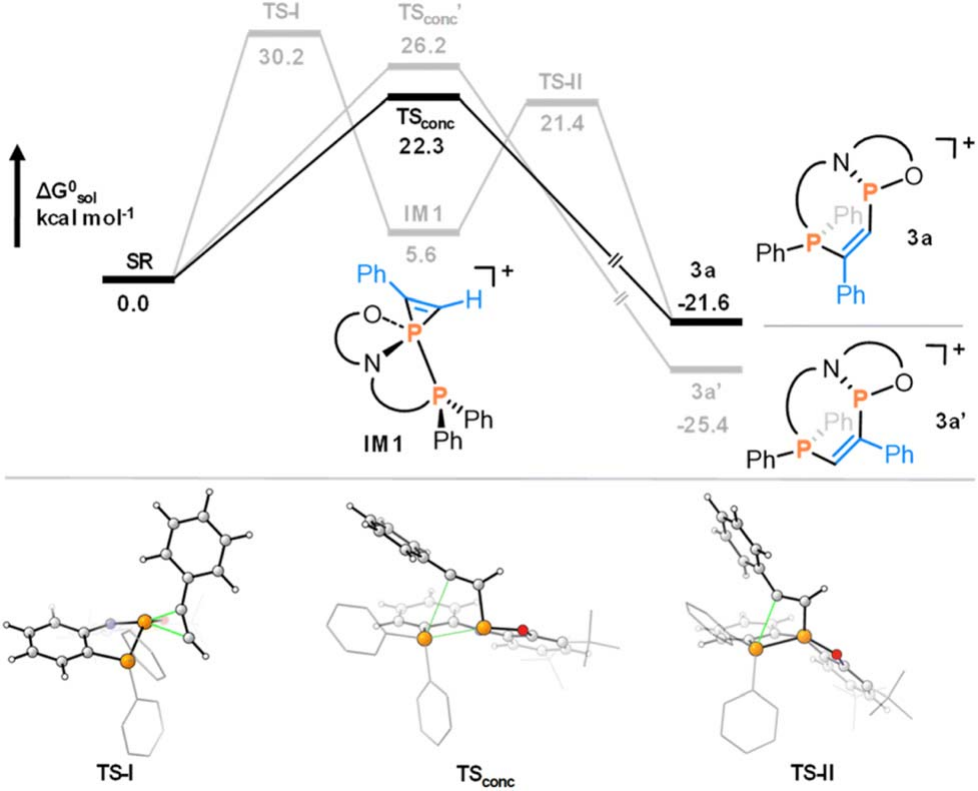
Calculated mechanism for the concerted reaction of phenylacetylene to 1. Reproduced from ref. 8.

Combining unusual electronics, ligand constraints, and weakly-coordinating anions within a single molecular system is a powerful and creative strategy for challenging bond activation at main-group centres. A slew of recent contributions,^11,14,28–30^ including the subject of this Focus article, illustrate how bridging the established fields of pincer coordination chemistry and Lewis acid chemistry represents a rich vein for both fundamental and applied discoveries. Although the reactivity of 1 reported so far is stoichiometric, the fundamental principles it illustrated may lead to new catalytic strategies for transforming weakly-polar bonds.

## Author contributions

TJH and SSC co-wrote the manuscript.

## Conflicts of interest

There are no conflicts to declare.
